# Foxq1 promotes metastasis of nasopharyngeal carcinoma by inducing vasculogenic mimicry via the EGFR signaling pathway

**DOI:** 10.1038/s41419-021-03674-z

**Published:** 2021-04-19

**Authors:** Yunfan Luo, Jie Wang, Fan Wang, Xiong Liu, Juan Lu, Xiaoxiao Yu, Xuemin Ma, Xiaohong Peng, Xiangping Li

**Affiliations:** 1 Otorhinolaryngology Head and Neck Surgery, Nanfang Hospital of Southern Medical University, Guangzhou, China; 2 Otorhinolaryngology Head and Neck Surgery, Guangzhou Women and Children’s Medical Center, Guangzhou, China

**Keywords:** Head and neck cancer, Metastasis, Tumour angiogenesis, Drug delivery

## Abstract

In nasopharyngeal carcinoma (NPC), the treatment of tumor metastasis and recurrence is challenging and is associated with poor clinical efficacy. Vasculogenic mimicry (VM) is a new blood-supply model of malignant tumor that is closely related to tumors’ distant metastasis. Our previous study demonstrated that miR-124 could target Foxq1 to inhibit NPC metastasis. Whether Foxq1 affects metastasis through vasculogenic mimicry is worth consideration. In this study, we show that VM formation positively correlates with the expression of Foxq1, and EGFR, and the TNM stage in 114 NPC patient samples. Meanwhile, we show that VM-positive NPC patients have a poor prognosis. Furthermore, using in vitro and vivo approaches, we confirm that Foxq1 has a significant effect on NPC metastasis through promoting VM formation, which could be effectively inhibited by EGFR inhibitors (Nimotuzumab or Erlotinib). Also a synergistic efficacy of anti-EGFR and anti-VEGF drugs has been found in NPC inhibition. Mechanistically, the luciferase reporter gene and CHIP assays show that Foxq1 directly binds to the EGFR promoter region and regulates EGFR transcription. In conclusion, our results show that Foxq1 is regulated by miR-124 and that it promotes NPC metastasis by inducing VM via the EGFR signaling pathway. Overall, these results provide a new theoretical support and a novel target selection for anti-VM therapy in the treatment of nasopharyngeal carcinoma.

## Introduction

Nasopharyngeal carcinoma is a malignant tumor that has a high incidence in Southeast Asia, especially in southern China. Owing to the significant improvements in our understanding of the pathogenesis of nasopharyngeal carcinoma and the progress that has been made in diagnosis and treatment technologies, the survival rate of nasopharyngeal carcinoma has been significantly improved^1,2^. However, recurrence or distant metastasis is still the biggest challenge in the treatment of nasopharyngeal carcinoma, and the main cause of patients’ death^3^. Hence, clarifying the metastatic mechanism of NPC is key in improving the survival rate of NPC patients.

Angiogenesis is an essential process of malignant tumors’ growth and metastasis^4^. For a long time, angiogenesis was believed to be the only blood-supply model for tumors; Thus, anti-angiogenic therapy was considered as an effective treatment for preventing metastasis. Currently, anti-angiogenic drugs mainly target the VEGF/VEGFR signaling pathway, however, anti-VEGF drugs have not achieved certain clinical effects in the treatment of nasopharyngeal carcinoma^5,6^. Therefore, it is particularly important to investigate the causes that are associated with the poor efficacy of anti-angiogenic drugs in nasopharyngeal carcinoma.

Vasculogenic mimicry (VM) is a novel blood-supply system that was first proposed in invasive human melanoma by Maniotis et al. in 1999^7^. In 2016, the journal Science noted that vasculogenic mimicry supplement traditional angiogenesis in supplying nutrients to tumors^8^. The discovery of VM, which is a vascular network pattern formed by highly invasive tumor cells that replace endothelial cells, partly explained the resistance to anti-angiogenic drugs^9,10^. Previous reports have also preliminarily confirmed that single anti-angiogenic therapy were ineffective in inhibiting VM, which might impair the effectiveness of anti-angiogenic approaches^11–13^. Recently, increasing evidences indicated that VM exists in numerous solid tumors, including head and neck carcinoma, and breast, liver, ovarian, gastric, and prostate cancers^14^, and it played an important role in tumor metastasis^15^. In nasopharyngeal carcinoma, there were only few studies on vasculogenic mimicry^16,17^, while neither of these focused on VM role in NPC metastasis. VM-specific regulatory mechanism in nasopharyngeal carcinoma remains to be further explored, which may lead to the discovery of new therapeutic targets.

In a previous study, we have demonstrated that miR-124 could target Foxq1 to inhibit NPC metastasis^18^. However, the specific mechanism of NPC metastasis that was induced by Foxq1, remained elusive. Foxq1 is a transcription factor that belongs to the Fox family, and is one of the key regulators of multiple cellular functions and plays an important role in tumors’ development, invasion, and metastasis^19^. Recently, studies on Foxq1 oncogenic and metastatic roles, have received extensive attentions^20,21^. Although the relationship between Foxq1 and VM has not been reported, other studies have shown that Foxq1 promotes tumor metastasis by promoting epithelial-mesenchymal transition (EMT) in a variety of tumors, including gastric, lung, colorectal, breast, and bladder cancers^22–24^. Meanwhile, EMT was closely related to VM formation, and EMT-related transcription factors, such as Twist, Snail and ZEB1, were highly upregulated in VM forming tumor cells^25–27^. These findings demonstrated that EMT-related transcription factors play a significant role in VM formation. Therefore, whether foxq1 affects the metastasis of nasopharyngeal carcinoma by regulating VM is worthy of further study.

In this study, we found that the epidermal growth factor receptor (EGFR) is directly regulated by Foxq1. EGFR high expression has been shown to correlate with poor prognosis in NPC patients with undifferentiated nasopharyngeal carcinoma^28^. Unlike anti-angiogenic therapy, the combination of anti-EGFR drugs, such as Nimotuzumab, and Cetuximab, with chemoradiotherapy have been proved to maximize the survival of stage II-IVb NPC patients^29^. However, the exact mechanism of anti-EGFR therapy in the treatment of NPC is still unclear. Meanwhile, as EGFR has been reported as an important regulator of VM in various malignancies^30,31^, we considered that VM may be the main leading cause that is associated with the difference in efficacy of anti-angiogenic drugs and anti-EGFR drugs in nasopharyngeal carcinoma. Thus in this manuscript, we examined the effect of anti-EGFR drugs on VM formation and the combined effect of anti-EGFR and anti-angiogenic drugs in NPC.

In summary, our study aimed at clarifying whether Foxq1 promotes NPC metastasis through regulating VM formation and confirm the specific functional relationship between Foxq1 and EGFR in promoting VM formation. We also provide a novel theoretical support for anti-EGFR therapy and clarify the possible causes that are related to the poor efficacy of anti-VEGF drugs in NPC.

## Materials and methods

### Clinical samples

The NPC tissue specimens (*n* = 114) were obtained from the Nanfang Hospital of Southern Medical University (GuangZhou, China). They were collected by biopsy from NPC patients between 2007 and 2019, and pathologically confirmed as NPC. Detailed pathological, clinical data and survival durations of the NPC patients were collected for all samples through outpatient and telephone follow-ups. The control tissue specimens (*n* = 40) were collected from biopsy specimen of nasopharyngitis. Informed written consent was obtained from each patient. The TNM classification was performed according to the definitions of the seventh edition of the UICC-American Joint Committee on Cancer staging criteria. The use of these tissue samples was approved by the Ethics Committee of Southern Hospital of Southern Medical University.

### Immunohistochemical and CD31-PAS dual staining

Formalin-fixed, and paraffin-embedded tissues were sectioned at 4 mm thickness, then harvested and fixed in 4% paraformaldehyde overnight at 4^ o^C. The antigen blocking was performed using 10% goat serum (AR0009,Boster,China). The sections were probed overnight at 4^ o^C with primary anti-Foxq1 (PA5-40772, Invitrogen, USA), anti-CD31 (ab134168, Abcam, USA) and anti-EGFR (ZM-0093, Zsbio, China) antibodies. The staining was detected using the DAB system (ZLI-9017, Zsbio, China). To detect VM structures, a PAS staining kit (G1281, Solarbio, China) and anti-CD31 (ab28364, Abcam, USA) were used. The numbers of positive cells were counted from ≥5 randomly chosen fields and at ×400 magnification, by two independent pathologists.

### Cell culture

All NPC cells were acquired from the Cancer Research Center of Southern Medical University and cultured in RPMI-1640 medium (PM15101,Thermo Fisher Scientific, USA), supplemented with 10% fetal bovine serum (10270-106, Thermo Fisher Scientific, USA), 100 mg/ml streptomycin (15140-122, Thermo Fisher Scientific, USA), and 100 U/ml penicillin (15140-122, Thermo Fisher Scientific, USA), and maintained at 37 °C in a humidified atmosphere with 5% CO_2_.

### RNA isolation, reverse transcription, and quantitative real-time PCR

Total RNA was extracted from the samples using RNAiso Plus (R401-01, Vazyme, China) and reversely transcribed to cDNA using the HiScipt III RT SuperMix for Quantitative Real-time PCR (+gDNA wiper) (R323-01, Vazyme, China). The quantitative reverse transcription PCR (qRT-PCR) was performed using ChamQ SYBR qRT-PCR Master Mix (Low ROX Premixed) (Q331-02, Vazyme, China) on an ABI QuantStudio5 System. GAPDH was used as the mRNAs endogenous control. All samples were normalized to the internal control, and the relative expression levels were calculated using the relative quantification assay. The primer sequences for qRT-PCR are shown in Additional file 1.

### Western blot

The proteins were extracted from the samples using the radio immunoprecipitation assay lysis buffer (P0013B, Beyotime, China), containing a protease-inhibitor cocktail (HY-K0010, MCE, USA). The proteins were lysed in SDS-loading buffer (FD006, Fdbio, China), then the lysates were resolved on sodium dodecyl sulphate–polyacrylamide gel electrophoresis and transferred to polyvinylidene fluoride membrane (IPVH00010, Millipore, USA). The membrane was incubated with polyclonal antibodies against EGFR (AF6043, Affinity, China), phospho-EGFR (AF3047, Affinity, China), MMP2 (AF0577, Affinity, China), MMP9 (AF5228, Affinity, China), VE-Cadherin (AF6265, Affinity, China), AKT (bsm-33282M, Bioss, China), phospho-AKT (bs-0876R, Bioss, China), Foxq1 (PA1-31951, Invitrogen, USA) or GAPDH (AC033, Abclonal, China) at a dilution of 1:1000, then incubated with species-specific HRP-conjugated secondary antibodies at a dilution of 1:5000. The immunoreactive bands were visualized by enhanced chemiluminescence (WBKLS0100, Millipore, USA). phosphatase inhibitor cocktail (HY-K0021, MCE, USA) was used for the phosphoprotein blots, e.g., p-EGFR and p-AKT.

### Cell transfection

The full-length sequences of Foxq1 were amplified by PCR and cloned into the pCMV3 vector (HG20110-UT, Sino Biological, China) to construct pCMV3-Foxq1 overexpressing plasmid. Plasmids were transfected into the cells using the Lipofectamine 3000 transfection reagent (L3000008, Thermo Fisher Scientific, USA). Lentiviruses that overexpress miR-124 or Foxq1 knockdown short-hairpin RNA (Genechem, China) were used to infect NPC cell lines and according to the manufacture’s instruction.

### Dual-luciferase assay

Before transfection, 293T cells (1 × 10^5^ cells) were plated in a 24-well plate for 24 h and co-transfected with the control pcDNA3.1 plasmid (2.0 µg/ml) or the Foxq1 plasmid (2.0 µg/ml) together with the control vector pGL3 (2.0 µg/ml) or the EGFR plasmid (2.0 µg/ml) using Lipofectamine 3000 (L3000008,Thermo Fisher Scientific, USA). After 36 h incubation, cell lysates were prepared and firefly/renilla luciferase values were quantified using the Dual-Luciferase Reporter Assay System (E1910, Promega, USA) on a GloMax-96 plate reader (Promega).

### CHIP-PCR

CHIP experiment was performed using the EZ-Magna ChIP™ A/G (17-10086, Millipore, USA) and according to the manufacture’s instruction. Anti-Foxq1 antibodies (sc-166266, SantaCruz, USA) were used for immunoprecipitation and the bound DNA was detected using two primer sets that were designed against the human EGFR promoter (for the sequences see Additional file 1).

### Three-dimensional culture

A 24-well plate was coated with 100μl growth factor-reduced Matrigel (354230, BD Biosciences, USA), that was polymerized at 37 ^o^C for 1 h, after which 1 × 10^5^ cells that were suspended in 500 μl of medium containing 10% FBS, were plated on the surface of the gel and incubated at 37^ o^C for 24 h. Three wells were provided for each group. The cells were then photographed under an inverted microscope (IX71, OLYMPUS, Japan). The Statistical results of the average number of the tubular structures were counted by ImageJ.

### Immunofluorescence staining

The cells were fixed with 4% paraformaldehyde for 15 min, then blocked with 1% BSA at 37 ^o^C for 30 min and incubated with an Foxq1 mouse monoclonal antibody (H00094234-M05, Abnova, USA), or a rabbit polyclonal antibody against EGFR, MMP2, MMP9 or VE-Cadherin. Then, the cells were washed with PBS and incubated at room temperature for 1 h with a secondary antibody conjugated with Alexa Fluor 555 (A0453, Beyotime, China). Finally, the cells were incubated with a mounting medium (with DAPI) (S2110, Solarbio, China) at room temperature for 3 min and all samples were observed using a scanning microscope (BX63, OLYMPUS, Japan)

### Tumor xenograft model and tumor metastasis assay in vivo

Four to 5-week-old BALB/C nude mice were purchased from the Center of Laboratory Animal of Southern Medical University, and the animal protocol was approved by the Institutional Animal Care and Use Committee of Southern Medical University. The animals were grouped randomly. For the subcutaneous animal model, control and Foxq1 overexpressing 5–8F cells, were injected subcutaneously into the flank of BALB/c nude mice, respectively (1 × 10^6^ cells/mouse; *n* = 6/group). The tumor volumes were monitored every 3 days. Two weeks later, the mice were euthanized, and the tumors were dissected out for further analyses. For the in vivo tumor metastatic assay, we used the lung metastatic model. Control and Foxq1 overexpressing 5–8F cells were injected through tail vein into BALB/c nude mice, respectively (1 × 10^6^ cells/mouse; *n* = 6/group). Five weeks later, the mice were euthanized, and the lung metastatic nodules were counted. For the drug sensitivity experiment, Foxq1 overexpressing 5–8F cells were separately inoculated into the flanks or the tail vein of the BALB/C nude mice, and randomly divided into three groups (1 × 10^6^ cells/mouse; *n* = 6/group), and treated with Erlotinib (i.g., 50 mg/kg), Nimotuzumab (i.v., 20 mg/kg), or physiological saline, respectively. After the mice were euthanized, the xenograft tumors were collected, and the incidences of lung metastases were recorded.

### Statistical analyses

The SPSS 25.0 software was used for statistical analyses. All data were from at least three independent experiments. The two-tailed student’s *t*-test was used for comparing two independent groups. The two-tailed Mann–Whitney test was used to analyze the data with non-Gaussian distribution. The Gaussian distribution data was analyzed by Welch’s correction if variances, which was determined by the *F*-test, when significantly different. One-way ANOVA analysis of variance was used to compare multiple groups. The Kaplan–Meier method and log-rank test were used to construct survival curves. The relationship between Foxq1 and EGFR was analyzed using the Spearman’s correlation analysis. The data are shown as the mean ± SEM unless otherwise. *p*-values of <0.05 were considered statistically significant.

## Results

### Foxq1 expression is high in NPC and significantly correlates with VM formation

**Fig. 1:**
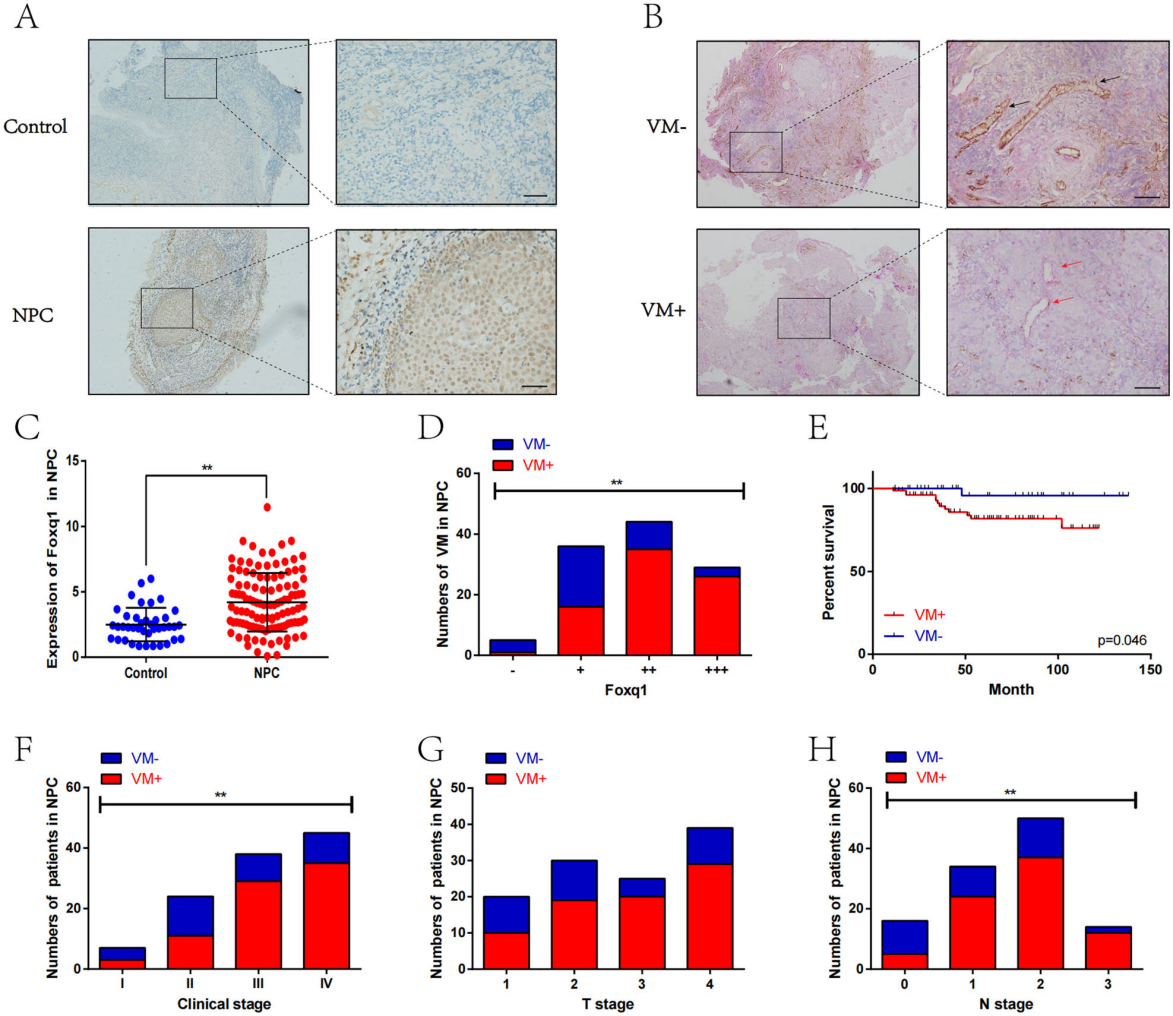
Foxq1 is highly expressed in NPC tissues, and with a significant correlation with VM formation. **A** Region showing typical Foxq1 expression in NPC and control; Scale bars represent 50 μm. **B** Typical morphology of VM (red arrow) and endothelial vessels (black arrow) in NPC visualized using CD31/PAS double staining; Scale bars represent 50 μm. **C** Statistical results of Foxq1 expression in NPC and control tissues; *p* < 0.001. **D** Relationship between Foxq1 grades and VM in NPC tissues; *p* < 0.001. **E** Kaplan–Meier survival curves between NPC-positive and negative VM groups; *p* = 0.046. **F**–**H** The relationship between VM and clinical stage (*p* = 0.013), T stage (*p* = 0.127) and N stage (*p* = 0.004) in NPC. Data are presented as mean ± s.d of three independent experiments.

Using IHC staining, Foxq1 expression was quantified in control and NPC tissues, containing 114 NPC patient samples (7, 24, 38, and 45 cases of grade I, II, III, and IV) and 40 nasopharyngitis samples. The results showed that Foxq1 was significantly upregulated in NPC tissues compared with nasopharyngitis tissues (*p* < 0.001) (Fig. 1A, C and Fig. S1). Thereafter, we assessed the VM vessels (CD31-negative, PAS-positive) in 114 NPC samples. Images of the VM typical morphology (red arrow) and endothelial vessels (black arrow) are shown in Fig. 1B. The immunohistochemical staining showed that 68% of the samples (78/114) were VM-positive, and 32% of the samples (36/114) were VM-negative. The statistical analysis indicated a strong correlation between VM and Foxq1 (*p* < 0.001) (Fig. 1D). Meanwhile, VM was significantly related with clinical stage (*p* = 0.013) (Fig. 1F) and N stage (*p* = 0.004) (Fig. 1H), but not with T stage (*p* = 0.127) (Fig. 1G), age (*p* = 0.662) or sex (*p* = 0.577) (Table 1). The result of the Kaplan–Meier analysis indicated that VM-positive NPC patients had shorter survival times than the VM-negative patients (*p* = 0.046) (Fig. 1E). Taken together, Foxq1 is highly expressed in NPC tissues, while VM, which potentially predicts poor prognosis in NPC patients, is clinically correlated with Foxq1 expression.

**Table 1:** The relationship between VM expression (VM + : CD31–/PAS + ; VM-: CD31 + /PAS–) and NPC clinicopathological characteristics.

		Total	VM+	VM−	*p*-value
	Cases (*n*)	114	78	36	0.577
Sex	Man	80	56	24	
	Female	34	22	12	
Age	<50	73	54	19	0.457
	≧50	41	24	17	
Foxq1	4.00(2.57–6.00)	4.75(3.00–6.54)	2.66(1.50–4.14)	<0.001	
EGFR	6.61 ± 0.24	7.36 ± 0.26	4.95 ± 0.40	<0.001	
TNM stage	I	7	3	4	0.013
	II	24	11	13	
	III	38	29	9	
	IV	45	35	10	
T stage	1	20	10	10	0.127
	2	30	19	11	
	3	25	20	5	
	4	39	29	10	
N stage	0	16	5	11	0.004
	1	34	24	10	
	2	50	37	13	
	3	14	12	2	
Foxq1	-	5	1	4	<0.001
	+	36	16	20	
	+ +	44	35	9	
	+ + +	29	26	3	
EGFR	-	3	1	2	<0.001
	+	18	5	13	
	+ +	56	39	17	
	+ + +	37	33	4	

### Foxq1 promotes VM formation in vitro

**Fig. 2:**
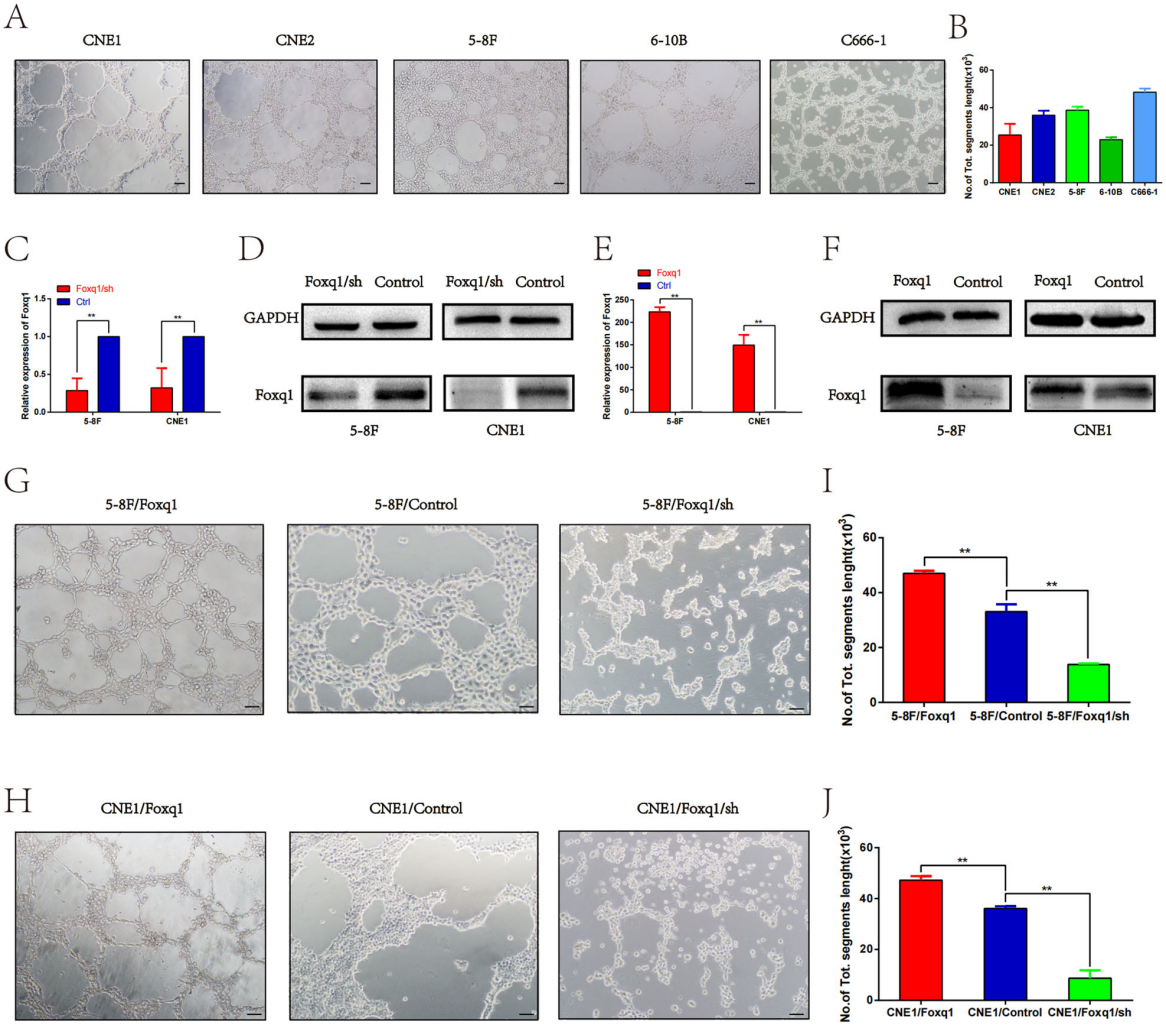
Foxq1 promotes VM formation in NPC cells. **A** VM channel formation in 3D culture of different types of NPC cells (magnification, ×200); Scale bars represent 50 μm. **B** The statistical results of the average numbers of tubular structures in NPC cells that was counted using ImageJ. **C**, **D** qRT-PCR (left) and western blot (right) of Foxq1 expression in Foxq1 knockdown 5–8F, and CNE1. **E**, **F** qRT-PCR (left) and western blot (right) of Foxq1 expression in Foxq1 overexpressing 5–8F and CNE1. **G**, **H** VM channel formation in Foxq1 dysregulated 5–8F, and CNE1 (magnification, ×200); Scale bars represent 50 μm. **I**, **J** The statistical results of the average number of tubular structures in Foxq1 dysregulated 5–8F, and CNE1 that was counted using ImageJ. Data are presented as mean ± SD of three independent experiments.

We first confirmed that NPC cell lines, including 5–8F, 6–10B, CNE1, CNE2 and C666-1, could form different degrees of vessel-like structures in 3D culture (Fig. 2A, B). It was obvious that poorly differentiated NPC cells (5–8F, CNE2, and C666-1) had a greater ability to form vessel-like structures compared to highly differentiated NPC cells (6-10B, CNE1), suggesting that the VM formation ability of NPC cells significantly correlates with the degree of malignancy. To investigate Foxq1 function in regulating VM formation, stably transfected NPC cell lines (5–8F, CNE1) that were knocked down or overexpressed Foxq1 were established. The expression of Foxq1 in these cells was investigated by qRT-PCR and western blot (Fig. 2C–F). A three-dimensional cell culture system was used to characterize the VM formation capacity of the dysregulated-Foxq1 cells. As Foxq1-depleted NPC cells exhibited a loss of VM formation capacity, the Foxq1 overexpressing cells could significantly promote VM formation (Fig. 2G–J). These results imply that Foxq1 contributes to VM generation in NPC cells.

### Foxq1 increased the expression of EGFR and VM-related genes, while Erlotinib inhibited Foxq1-induced VM formation

**Fig. 3:**
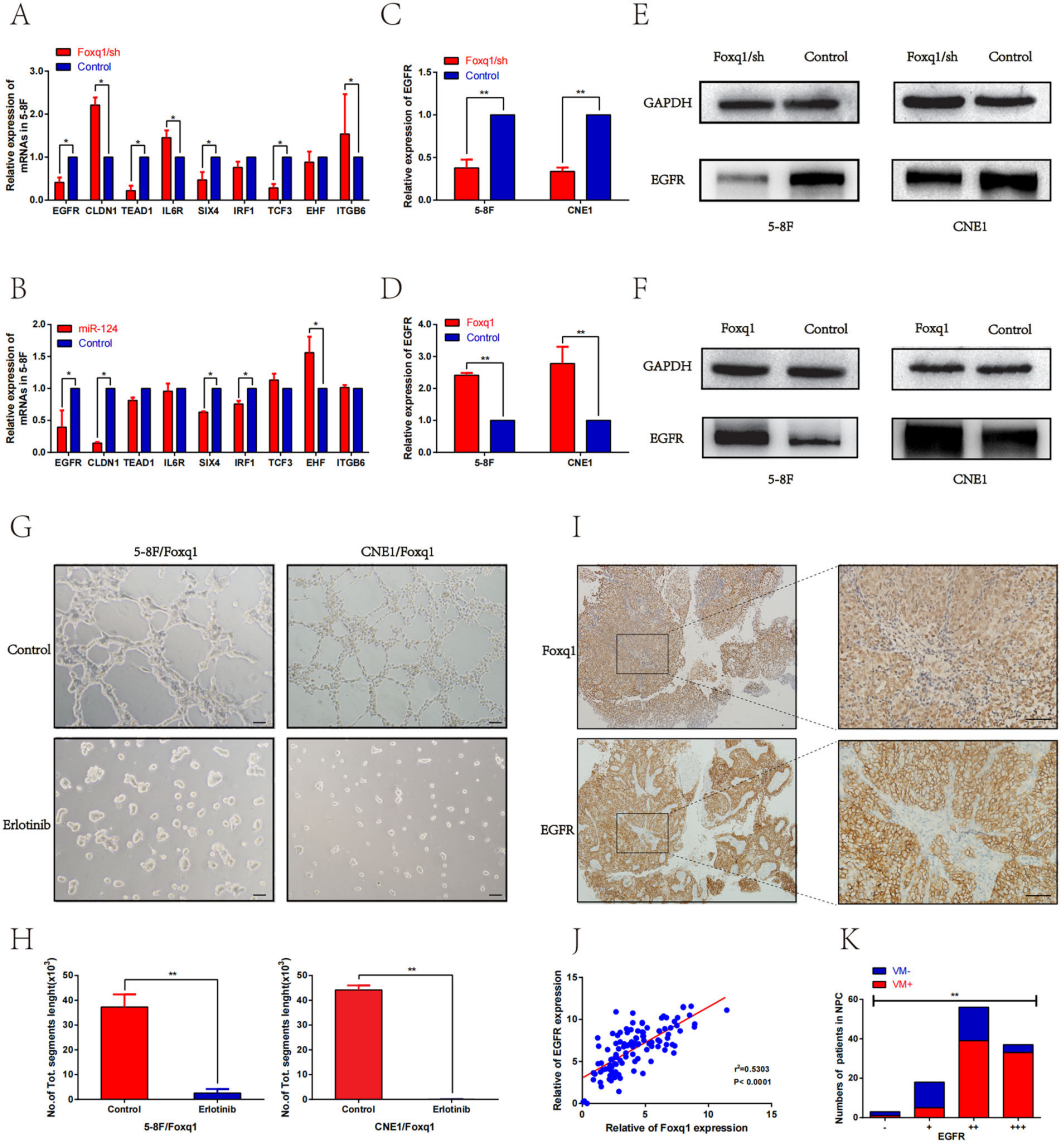
Foxq1 increases the expression of EGFR, while Erlotinib inhibits Foxq1-induced VM formation. **A**, **B** qRT-PCR was used to assess the expression of predicted genes in 5–8F cells that overexpressed miR-124 or had a Foxq1 knockdown. **C**–**F** qRT-PCR (left) and western blot (right) of EGFR expression in Foxq1 dysregulated 5–8F, and CNE1. **G** VM channel formation in 3D culture of cells overexpressing Foxq1 after treatment with Erlotinib (20 μM) or DMSO. **H** The statistical results of the average numbers of tubular structures in each group; *p* < 0.001. **I** Region showing typical expression of Foxq1 and EGFR in NPC tissues; Scale bars represent 50 μm. **J** Pearson correlation analysis of the association between Foxq1 and EGFR in NPC tissues (*r*² = 0.5303; *p* < 0.001). **K** Relationship between the grades of EGFR and VM in NPC tissues; *p* < 0.001. Data are presented as mean ± SD of three independent experiments.

Although Foxq1 expression positively correlates with VM, the exact mechanism of this event is still unclear. Using bioinformatics databases (Oncomine, GEO, TCGA), we selected some genes as miR-124 and Foxq1 possible targets. QRT-PCR was used to verify the expression of these genes in the 5–8F cell line that overexpressed miR-124 or the down-regulated Foxq1 (Fig. 3A, B). Hence, EGFR was chosen as a Foxq1 downstream target gene. Subsequently, qRT-PCR and western blot were used to assess the expression of EGFR in the 5–8f and CNE1 cell lines that overexpressed or had a Foxq1 knockdowns. The results showed that Foxq1 overexpression significantly promotes EGFR expression, while Foxq1 downregulation inhibits EGFR expression (Fig. 3C–F). To confirm whether Foxq1 exerts a VM promoting effect through EGFR in NPC cells, the Foxq1 overexpressing NPC cells were pre-treated with Erlotinib (EGFR inhibitor) for 24 h, then the capacity of VM formation was detected by 3D culture. As expected, Erlotinib markedly abrogated the promotion of VM formation that was induced by Foxq1 (Fig. 3G, H). The above results confirmed that Foxq1 promotes VM formation through regulating EGFR expression in vitro. In NPC tissues, the Pearson correlation analysis showed a significant correlation between Foxq1 and EGFR (*p* < 0.001, *r*² = 0.5303) (Fig. 3I, J), and that EGFR significantly correlates with VM (*p* < 0.001) (Fig. 3K).

**Fig. 4:**
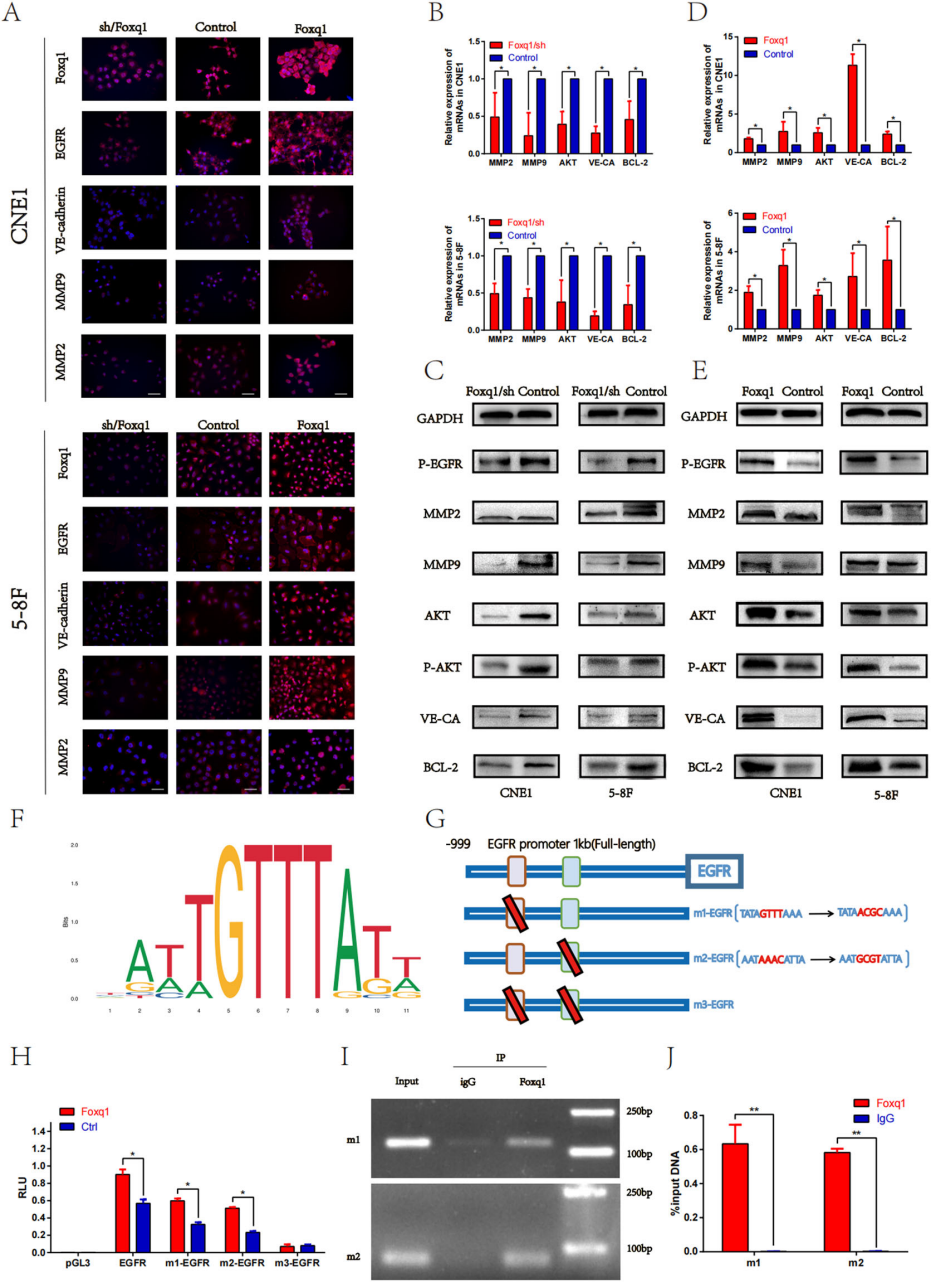
Foxq1 promotes the EGFR signaling pathway and VM-related genes through a direct binding to the EGFR promoter. **A** Immunofluorescence staining of Foxq1,EGFR,VE-cadherin,MMP2, and MMP9 in Foxq1 dysregulated 5–8F, and CNE1; Scale bars represent 50 μm. qRT-PCR (above) and western blot (down) were used to monitor the expression of EGFR signaling pathway and VM-related genes in 5–8F, and CNE1 after Foxq1 downregulation (**B**, **C**) or overexpression (**D**, **E**). **F** The binding motif of Foxq1 from the JASPAR database. **G** A diagram showing the relationship of full-length (FL) and mutant EGFR promoters. **H** 293 T cells were transfected with Foxq1 overexpressing or control vector. The luciferase reporter gene vectors carrying FL or EGFR promoter mutant were co-transfected respectively for 36 h. Then, the dual-luciferase activity was detected. **I** The CHIP-PCR assay was used to assess the binding of Foxq1 to the EGFR promoter region. **J** Anti-Foxq1-pulled down chromatins were analyzed by qRT-PCR. Data are presented as mean ± SD of three independent experiments.

Furthermore, qRT-PCR, western blot, and immunofluorescence staining were used to evaluate Foxq1 effect on the EGFR signaling pathway and VM-related genes. The results showed that the expression of genes, such as AKT, p-AKT, BCL-2, MMP2, MMP9, and VE-cadherin, significantly and positively correlate with Foxq1 expression (Fig. 4A–E). These results support our hypothesis that Foxq1 is involved in VM formation trough the EGFR signaling pathway in NPC.

### Foxq1 is a direct transcriptional regulator of EGFR

To further explore the specific interaction between Foxq1 and EGFR, we used UCSC and JASPAR databases to predict Foxq1-binding sites in the EGFR promoter. Multiple binding sites were predicted (Fig. 4F)^32^ and two most likely binding sites were selected and verified by the luciferase reporter assay. First, we cloned the full-length (FL) EGFR promoter (1 kb) or the mutant EGFR promoter into the luciferase reporter plasmids. The mutant EGFR promoters contained three degenerate Foxq1-binding elements (TBEs), such as ATAGTTTAAA (m1), AATTAAACATTA (m2) and both of them (m3), respectively, (Fig. 4G). Then, the luciferase reporter plasmids were co-transfected with Foxq1 or control plasmids into 293T cells, and the fluorescence signal was detected after 36 h. The result showed an increased transactivation of EGFR in the Foxq1 group compared with the control group. Furthermore, mutant m1 or m2 reduced the EFGR transactivation, while mutant m3 had no significant binding activity with Foxq1 (Fig. 4H). These results indicated that m1 and m2 sites are the main Foxq1-binding sites in the EGFR promoter region. Next, we performed a chromatin immunoprecipitation (CHIP) assay to substantiate the interaction between Foxq1 and EGFR. CHIP-PCR showed that the anti-Foxq1 antibody successfully pulled down the predicted EGFR binding sequence (Fig. 4I). qRT-PCR analysis of the pulled down chromatins by Foxq1 and IgG antibodies implicated an enrichment of Foxq1 occupancies at the EGFR promoter region in the precipitated material with the Foxq1-specific antibody, but not with the IgG antibodies (Fig. 4J). These results support the possibility that Foxq1 directly regulates EGFR transcription. In conclusion, Foxq1 promotes EGFR transcription through its binding to the EGFR promoter m1 and m2 sites.

### Foxq1 promotes VM formation, and NPC growth and metastasis in vivo

**Fig. 5:**
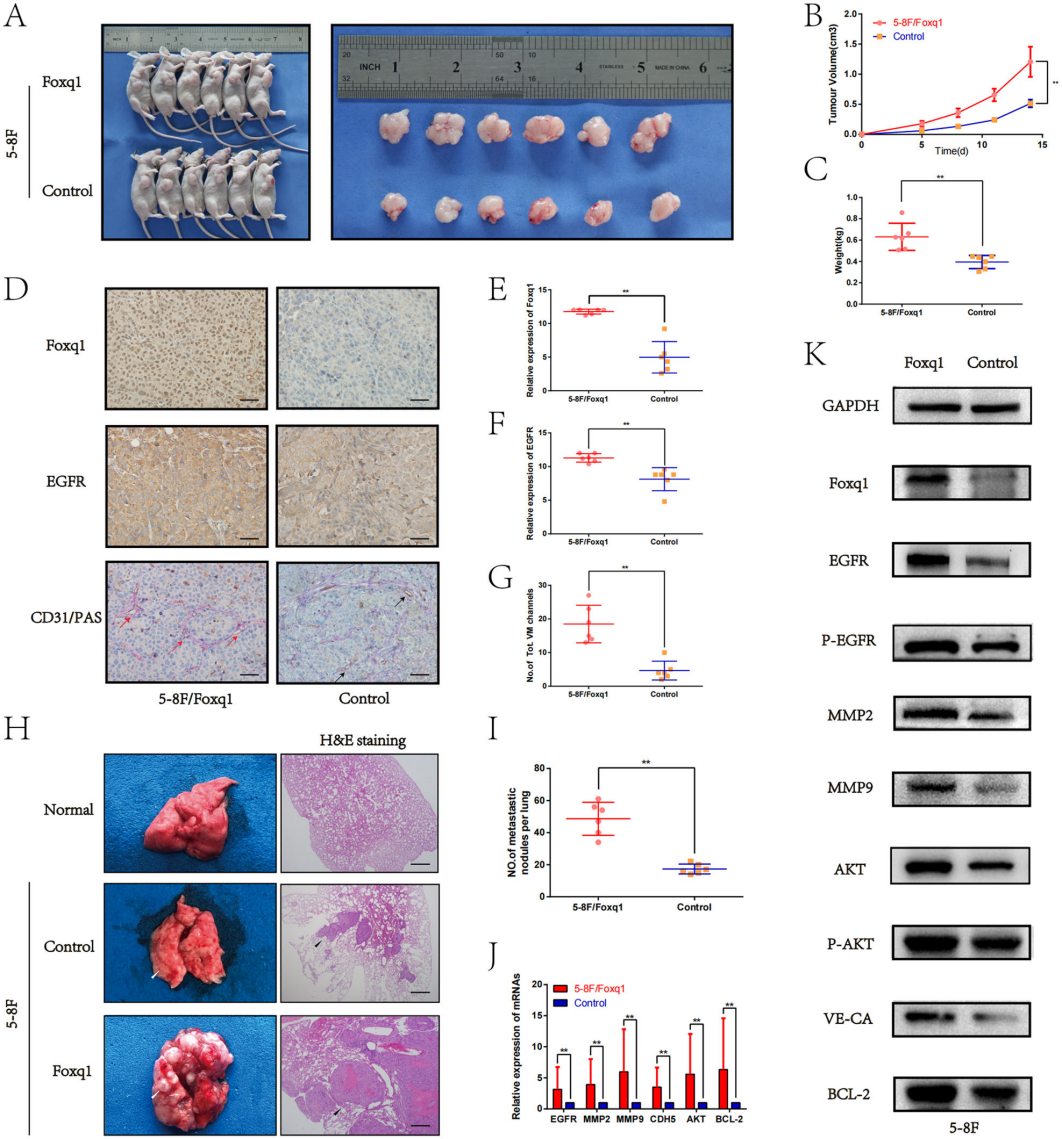
Foxq1 promotes VM formation, and NPC growth and metastasis through regulating EGFR in vivo. **A** Image of xenograft tumors from nude mice that were injected with Foxq1 overexpressing or control 5–8F cells (after 2 weeks). **B** The growth curves of xenograft tumors from each group; *p* < 0.001. **C** The weight of xenograft tumors from each group; *p* < 0.001. **D** IHC of Foxq1, EGFR and PAS/CD31 double staining of xenograft tumors from each group. Red arrows = VM; Black arrows = endothelial vessels; Scale bars represent 50 μm. **E**–**G** Statistical results of Foxq1(*p* < 0.001),and EGFR (*p* < 0.001) expression and VM channels (*p* < 0.001) in xenograft tumors from each group. **H** Metastatic nodules by tail vein injection of each group and normal lung (left). Image of H&E staining of lung sections from each group (right); Scale bars represent 500 μm. **I** Statistical results of the metastatic nodules in each group; *p* < 0.001. The expression of related genes in xenograft tumors from each group were monitored by qRT-PCR (**J**) and western blot (**K**). Data are presented as mean ± SD of three independent experiments.

We investigated in vivo the relationship between Foxq1, NPC growth and VM formation using a subcutaneous BALB/c nude mice xenograft tumor model (*n* = 6/group). Control and Foxq1stably overexpressing 5–8F cells were subcutaneously injected into BALB/c-nude mice, respectively. Through plotting the tumor growth curves, we found that tumors’ volume in the Foxq1-overexpressing group was significantly higher than that in the control group (Fig. 5A, B). Meanwhile, the tumors’ weight in the Foxq1-overexpressing group was significantly higher than that in the control group (Fig. 5C). The histologic analysis of the tumors indicated that the number of the VM events and the expression of EGFR, were significantly increased in the Foxq1-overexpressing group compared with those in the control group (Fig. 5D–G). Consistent with the Foxq1 tumor-promoting effects, qRT-PCR and western blot showed that the expression of related genes in the tumors were also dramatically increased in Foxq1-overexpressing group compared to those in the control group (Fig. 5J, K). These results show that Foxq1 promotes VM formation and NPC growth by increasing EGFR expression in vivo.

Moreover, an in vivo lung metastasis model was chosen to further verify Foxq1 role in NPC metastasis. The control and Foxq1 overexpressing 5–8F cells were inoculated into BALB/c-nude mice through tail vein, respectively. Five weeks later, their metastatic capacity was assessed through counting metastatic nodules in the lung. The results showed that the mice that were injected with Foxq1 overexpressing 5–8F cells formed significantly more metastatic nodules compared with the control mice (Fig. 5H, I). These results demonstrate that Foxq1 promotes NPC metastasis in vivo.

### In vivo, Erlotinib and Nimotuzumab inhibit Foxq1-induced VM formation, and NPC growth and metastasis

**Fig. 6:**
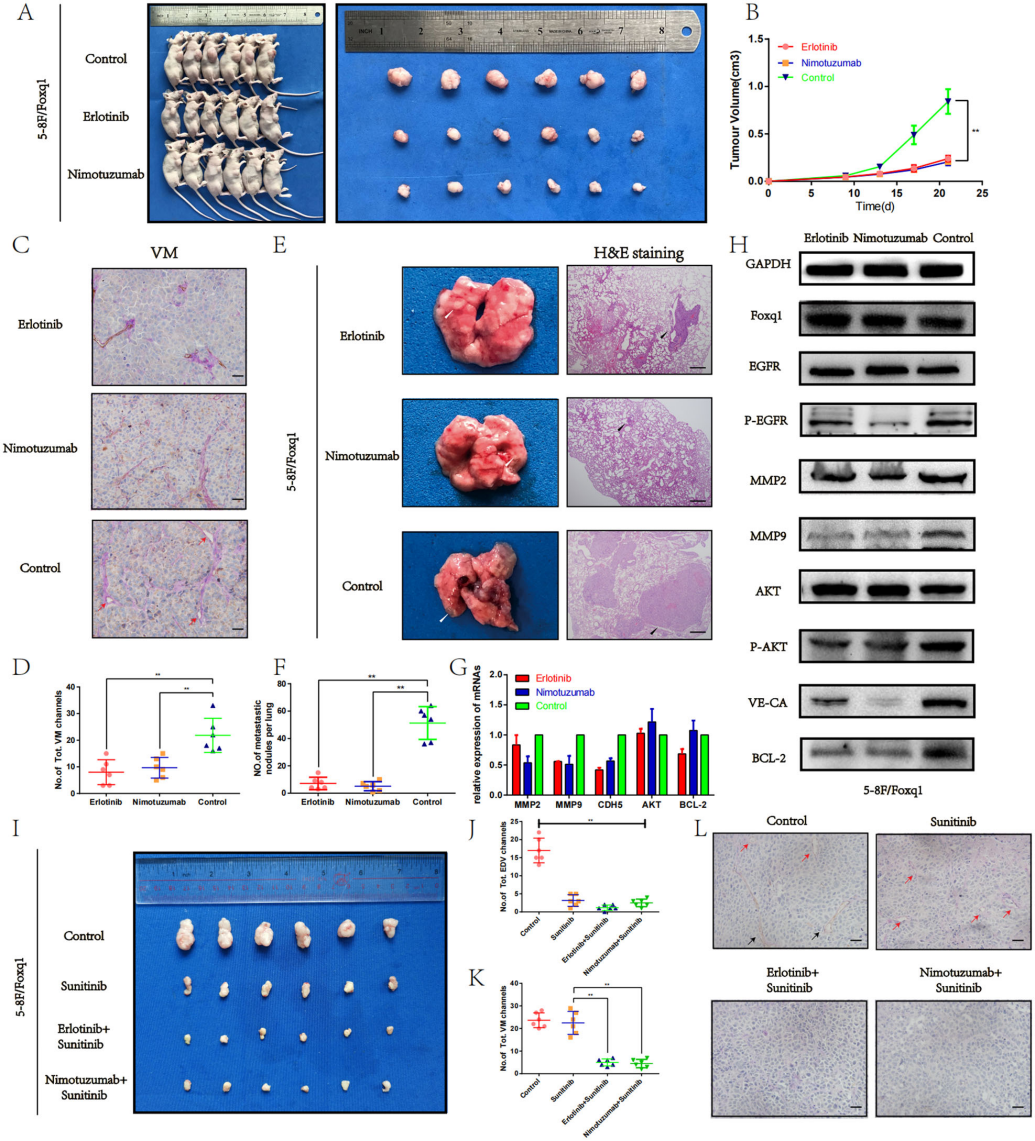
Erlotinib and Nimotuzumab could inhibit Foxq1-induced VM formation and NPC growth and metastasis in vivo. **A** Image of xenograft tumors from nude mice injected with Foxq1 overexpressing 5–8F cells, that were treated with Erlotinib (i.g,50 mg/kg), Nimotuzumab (i.v,20 mg/kg), or physiological saline, respectively. **B** The growth curves of the xenograft tumors from each group; *p* < 0.001. **C** PAS/CD31 staining of xenograft tumors from each group; Scale bars represent 50 μm. **D** Statistical results of VM channels in xenograft tumors from each group; *p* < 0.001. **E** Metastatic nodules in each group following tail vein injection (left). Image of H&E staining of lung sections from each group (right); Scale bars represent 500 μm. **F** Statistical results of metastatic nodules of each group; *p* < 0.001. The expression of related genes in xenograft tumors from each group were monitored by qRT-PCR (**G**) and western blot (**H**). **I** Image of xenograft tumors from nude mice injected with Foxq1 overexpressing 5–8F cells, that were treated with Sunitinib (i.g., 40 mg/kg), Sunitinib (i.g., 40 mg/kg) + Erlotinib (i.g., 50 mg/kg), Sunitinib (i.g., 40 mg/kg) + Nimotuzumab (i.v., 20 mg/kg), or physiological saline, respectively. **J** Statistical results of EDV channels in xenograft tumors from each group; *p* < 0.001. **K** Statistical results of VM channels in xenograft tumors from each group; *p* < 0.001. **L** PAS/CD31 staining of xenograft tumors from each group; Scale bars represent 50 μm. Data are presented as mean ± SD of three independent experiments.

To further investigate the effect of EGFR on Foxq1-induced VM, and NPC growth and metastasis in vivo, we injected the Foxq1 overexpressing 5–8F cells into BALB/c-nude mice subcutaneously or through the tail vein, then treated them with Erlotinib, Nimotuzumab or physiological saline, respectively. As expected, the tumors that were treated with Erlotinib or Nimotuzumab grew slower than those in the control group (Fig. 6B), while the volume and weight were also smaller than those in the control group (Fig. 6A and Fig. S2). Consistent with these results, IHC of the tumors using a PAS/CD31 double staining indicated that the number of VM events was significantly decreased in the Erlotinib or Nimotuzumab group compared with that of the control group (Fig. 6C, D). These results were further confirmed by investigating the expression of related genes that were assessed by qRT-PCR and western blot (Fig. 6G, H). In the lung metastasis model, the mice that were treated with Erlotinib and Nimotuzumab formed significantly less metastatic nodules compared that to the mice that were treated with physiological saline (Fig. 6E, F). In summary, these results demonstrate that, as the EGFR inhibitors, Erlotinib and Nimotuzumab, prevent Foxq1 from promoting VM formation, and NPC growth and metastasis.

### Combination with anti-EGFR and anti-VEGF drugs resulted in improved antitumor efficacy

Above results have revealed that anti-EGFR therapy is an effective strategy in inhibiting Foxq1-induced VM formation. While anti-VEGF therapy, which was used to prevent endothelium-dependent vessels (EDV), has been reported to have no inhibitory effect on VM^13^. Hence, it is worth to explore the combined effect of anti-EGFR therapy and anti-VEGF therapy. One week after Foxq1 overexpressing 5–8F cells were injected into BALB/c-nude mice subcutaneously, we treated them with Sunitinib, combination of Sunitinib and Erlotinib, or combination of Sunitinib and Nimotuzumab, respectively (Fig. S3). Although Sunitinib showed effective inhibition of tumors, the combination of anti-EGFR and Sunitinib groups grew slower than those in the group treated Sunitinib alone (Fig. 6I). The volume and weight (Figs. S4 and S5) of the combination treatment groups were also smaller than those in the control group or treated Sunitinib alone. Through CD31-PAS dual staining, we found that Sunitinib had strong inhibitory effect on angiogenesis, but no obviously effect on VM, While the combination of anti-EGFR and Sunitinib showed a significantly negative effect on both EDV and VM (Fig. 6J–L). These results confirmed the synergistic effect of anti-VEGF and anti-EGFR drugs, providing a new therapeutic option for nasopharyngeal carcinoma.

### Mir-124 inhibits the expression of EGFR and VM formation, events that are rescued by Foxq1

**Fig. 7:**
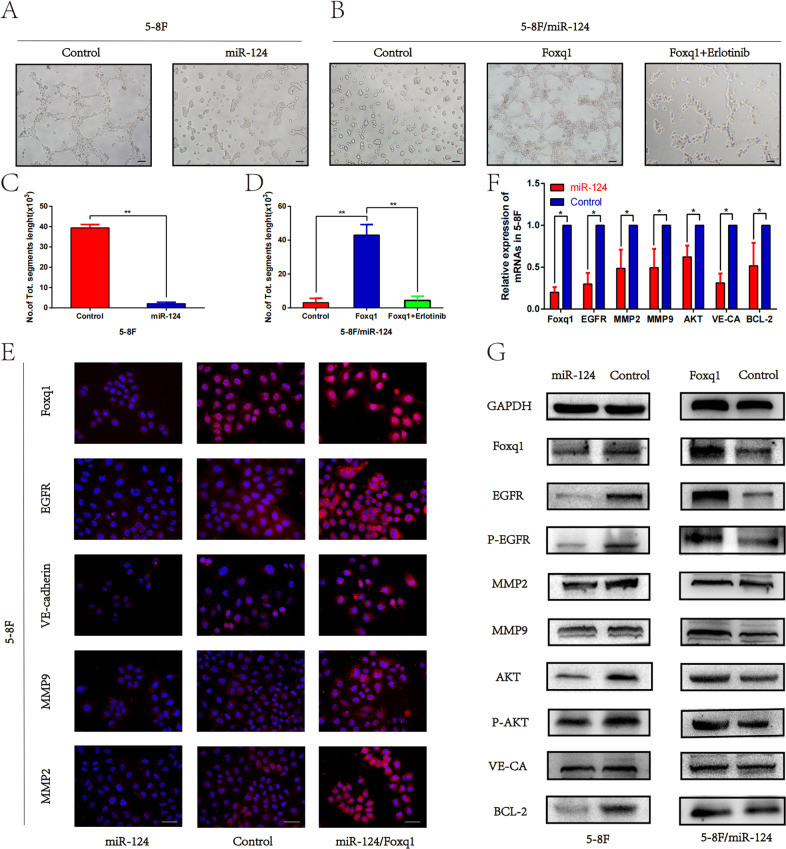
MiR-124 inhibits the EGFR signaling pathway and VM formation, that could be rescued by Foxq1 expression. **A** VM channel formation in miR-124 overexpressing and control 5–8F cells (magnification, ×200); Scale bars represent 50 μm. **B** VM channel formation in 5–8F cells that overexpressed both Foxq1 and miR-124; *p* < 0.001. Foxq1 could significantly reverse the inhibitory effect of miR-124 on VM formation, which could be inhibited by Erlotinib (magnification, ×200); Scale bars represent 50 μm. **C**, **D** The statistical results of the average number of tubular structures in each group. **E** Immunofluorescence staining of Foxq1, EGFR, VE-cadherin, MMP2 and MMP9 in 5–8F cells that overexpressed miR-124, control or simultaneously Foxq1 and miR-124, respectively; scale bars represent 50μm. qRT-PCR (**F**) and western blot (**G**) were used to monitor the expression of EGFR signaling pathway and VM-related genes in 5–8F cells that overexpressed miR-124, control or simultaneously Foxq1 and miR-124, respectively. Data are presented as mean ± SD of three independent experiments.

**Fig. 8:**
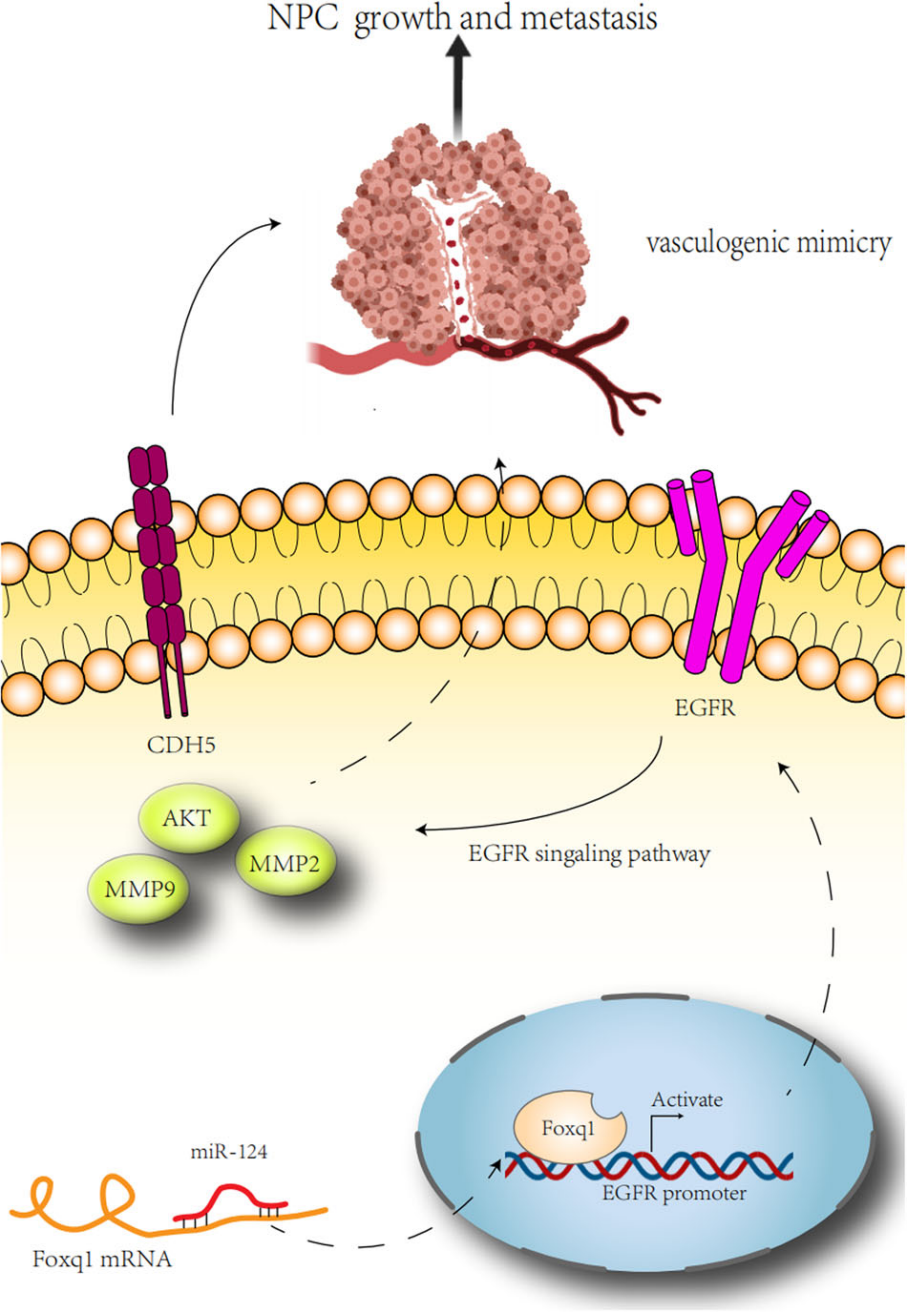
Schematic diagram of the regulation of VM formation and NPC metastasis by the miR-124/Foxq1 axis. Foxq1, which is the target of miR-124, could directly binds to the EGFR promoter and regulates EGFR transcription, which further promotes NPC growth and metastasis through upregulating EGFR signaling pathway and VM-related genes.

Our previous study confirmed that miR-124 could target Foxq1 to inhibit NPC growth and metastasis. In this study, we verified the VM formation capacity of the miR-124 overexpressing 5–8F cells and found that it was significantly reduced compared with the control group in the 3D cultures assay (Fig. 7A, C). To investigate the role of Foxq1 and EGFR in miR-124-induced VM inhibition, we conducted rescue experiments by overexpressing Foxq1 in 5–8f cells that also overexpressed miR-124. The results showed that Foxq1 could reverse the inhibitory effect of miR-124 on VM formation, and that was also inhibited by Erlotinib (Fig. 7B, D). Furthermore, we found that miR-124 overexpression significantly reduces the expressions of Foxq1, and EGFR signaling pathway and VM-related genes as demonstrated using qRT-PCR, western blot and Immunofluorescence staining (Fig. 7E–G). These data strongly support our hypothesis that the miR-124/Foxq1 axis inhibits VM formation through the EGFR signaling pathway (Fig. 8).

## Discussion

During tumorigenesis, oxygen and nutrients that are provided by blood, are indispensable. When reaching a diameter of ≥2 mm, the tumor needs a new blood-supply to meet its basic metabolic requirements^33^. Vasculogenic mimicry is surrounded by highly invasive tumor cells that are PAS-positive and CD31-negative staining highly invasive tumor cells, and provides sufficient blood-supply for tumor tissues^8^. Owing to this unique structure, tumor cells are in direct contact with the blood, which greatly increases the risk of distant blood metastasis^34^. Hence, VM frequently correlates with an advanced tumor grade, invasion, metastasis and poor prognosis^35^.

Our study confirmed the presence of vasculogenic mimicry in nasopharyngeal carcinoma tissues using a CD31-PAS dual staining. VM was significantly related to clinical stage, and the Kaplan–Meier analysis indicated that patients who are VM positive had a shorter survival time. Furthermore, vasculogenic mimicry in nasopharyngeal carcinoma was further detected in vitro and in vivo. These results indicated that VM is a poor prognostic indicator in nasopharyngeal carcinoma patients and that it plays an important role in its development.

Currently, the mechanism of VM regulation is controversial. Multiple molecular mechanisms, especially vascular endothelial (VE)-cadherin, erythropoietin-producing hepatocellular receptor A2 (EphA2), phosphatidyl inositol 3-kinase (PI3K), matrix metalloproteinases (MMPs), vascular endothelial growth factor receptor (VEGFR1), and hypoxia inducible factor (HIF)-1α, have been reported to participate in VM formation that is associated with tumor migration and invasion^36,37^. In addition, hypoxia, cancer stem cells (CSCs) and epithelial-mesenchymal transition (EMT) are regarded as significant factors in VM formation and tumor metastasis^38^. However, the exact mechanism of VM formation is still unclear and requires further studies.

In this study, we found that Foxq1 promotes EGFR expression at mRNA and protein levels, and that EGFR inhibitor drugs could inhibit Foxq1-induced VM formation. In vivo, Foxq1 promoted the growth and metastasis of nasopharyngeal carcinoma, which could be prevented by EGFR inhibitors. Meanwhile, the EGFR signaling pathway and VM, were significantly inhibited in the group that was treated with EGFR inhibitor drugs. Furthermore, Luciferase reporter gene and CHIP assays showed that Foxq1 directly binds to the EGFR promoter region and regulates its transcription. The above results indicate that Foxq1 is regulated by miR-124, which promoted NPC metastasis by inducing VM via the EGFR signaling pathway.

EGFR could activate a series of downstream signaling pathways to exert corresponding biological effects, such as cell proliferation, apoptosis and metastatic spread^39^. Meanwhile, EGFR has been proved to be overexpressed and acted as a poor prognostic factor in a variety of tumors, including head and neck, breast, lung, and colorectal cancers^40^; hence, EGFR drugs are widely used in cancer therapy. In nasopharyngeal carcinoma, EGFR expression rate was as high as 90%, and it was closely related to chemoresistance, radioresistance and poor prognosis^41^. Owing to its critical role, anti-EGFR therapy is considered to be an effective target in NPC treatment^42^, and therefore, it is necessary to clarify the specific mechanism of anti-EGFR targeted drugs in the treatment of nasopharyngeal carcinoma.

The mechanism of EGFR overactivation during tumor pathogenesis is mainly associated with the overexpression of EGFR or its ligands^43^. For example, the long noncoding RNA, EGFR-AS1, was shown to directly binds EGFR mRNA, which promotes cell growth and metastasis through upregulating EGFR expression in renal cancer^44^. While a recent study has shown that CD317 is associated with lipid rafts, and activates EGFR in hepatocellular carcinoma (HCC) cells by regulating its localization on the plasma membrane^45^. In this study, we demonstrated that Foxq1 directly binds to the EGFR promoter region and regulates its transcription. Furthermore, VM was regulated by the miR-124-Foxq1-EGFR axis in nasopharyngeal carcinoma. The above results illustrated that the inhibition of VM formation by the miR-124-Foxq1-EGFR axis might be the new mechanism of anti-EGFR drugs in the treatment of nasopharyngeal carcinoma.

Our results also indicated that the presence of VM may be a possible cause of anti-VEGF drugs’ failure in achieving satisfactory results in nasopharyngeal carcinoma treatment. Researchers have confirmed that effective therapeutic strategies should simultaneously inhibit VM and EDV^46^. Meanwhile, encouraging results has also been observed in clinical trials using a combination of VEGF and EGFR blockade^47^. For example, in non-small-cell lung cancer, the combination of Bevacizumab and Erlotinib has been confirmed to improve progression-free survival compared with erlotinib alone in a phase 3 trial^48^. In addition, the combination of Bevacizumab and Erlotinib has been shown to be in the treatment of metastatic renal cell carcinoma and special types of advanced breast cancer^49,50^. A randomized phase II trial also showed that the combination of bevacizumab and cetuximab might be a sensible treatment strategy in metastatic colorectal cancer^51^. Evidence from these trials supported the feasibility and efficacy of developing a combined VEGF and EGFR inhibition therapy for patients with solid tumors. Based on our research about the synergistic effect of anti-VEGF and anti-EGFR drugs, we consider that the combination of anti-EGFR and anti-VEGF therapy is an effective therapeutic strategy for advanced or metastatic nasopharyngeal carcinoma.

## Conclusions

In conclusion, we provide evidence that the miR-124-Foxq1-EGFR axis contributes to NPC growth and metastasis through regulating VM formation. These results provide new targets and options for nasopharyngeal carcinoma anti-VM therapy. Meanwhile, we demonstrate that VM, as a poor prognostic factor for nasopharyngeal carcinoma, can be inhibited by Erlotinib and Nimotuzumab. These suggest that anti-EGFR therapy is an effective strategy in inhibiting VM formation and in preventing metastasis of nasopharyngeal carcinoma. Finally, the combination of anti-EGFR and anti-VEGF therapy shows better tumor inhibition than single anti-VEGF therapy, which is beneficial to optimize the clinical treatment of NPC.

## Supplementary information

Supporting Informaiton

## Data Availability

The datasets during the current study available from the corresponding author on reasonable request.
