# Supplementary material for: Foxq1 promotes metastasis of nasopharyngeal carcinoma by inducing vasculogenic mimicry via the EGFR signaling pathway
Source: Cell Death Dis. 2021 Apr 19;12(5):411. doi: 10.1038/s41419-021-03674-z (PMC8055972; doi:10.1038/s41419-021-03674-z)
Supplement: Supplementary file 1 — Supporting Informaiton [file 41419_2021_3674_MOESM1_ESM.docx]

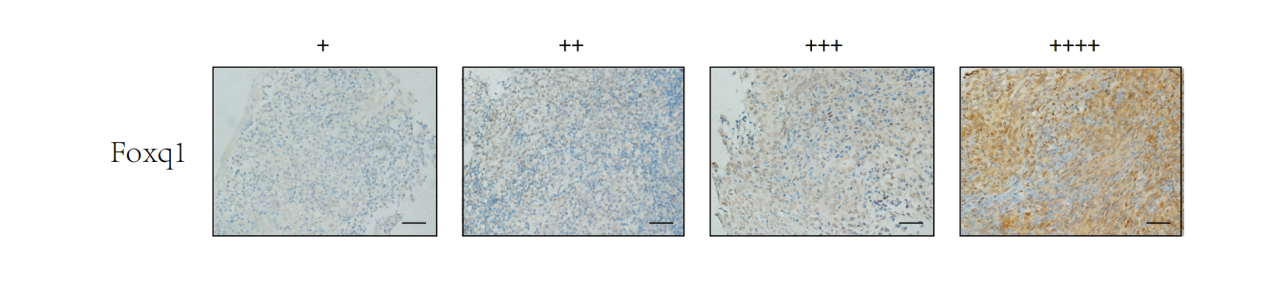


**Figure S1**. Typical images of Foxq1 with different ratings; Scale bars represent 50μm.


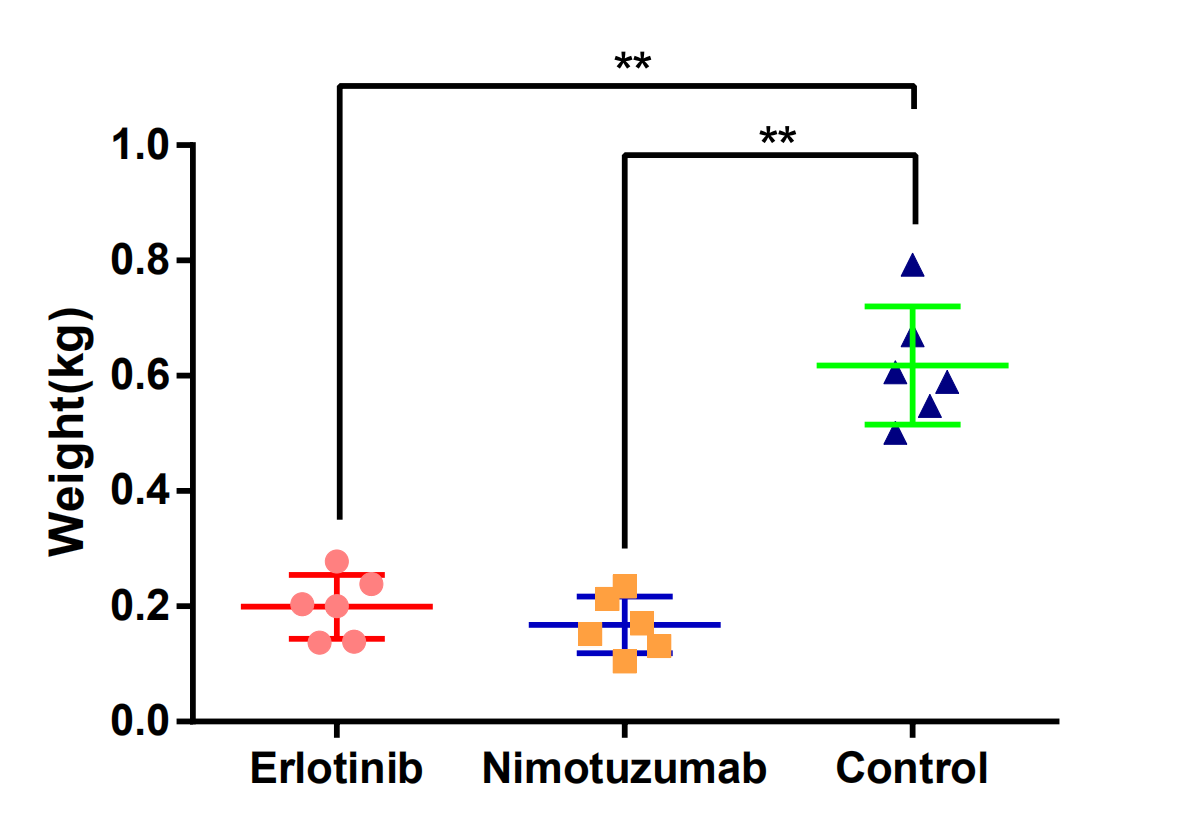


**Figure S2**. The weight of xenografts from each group; p<0.001.


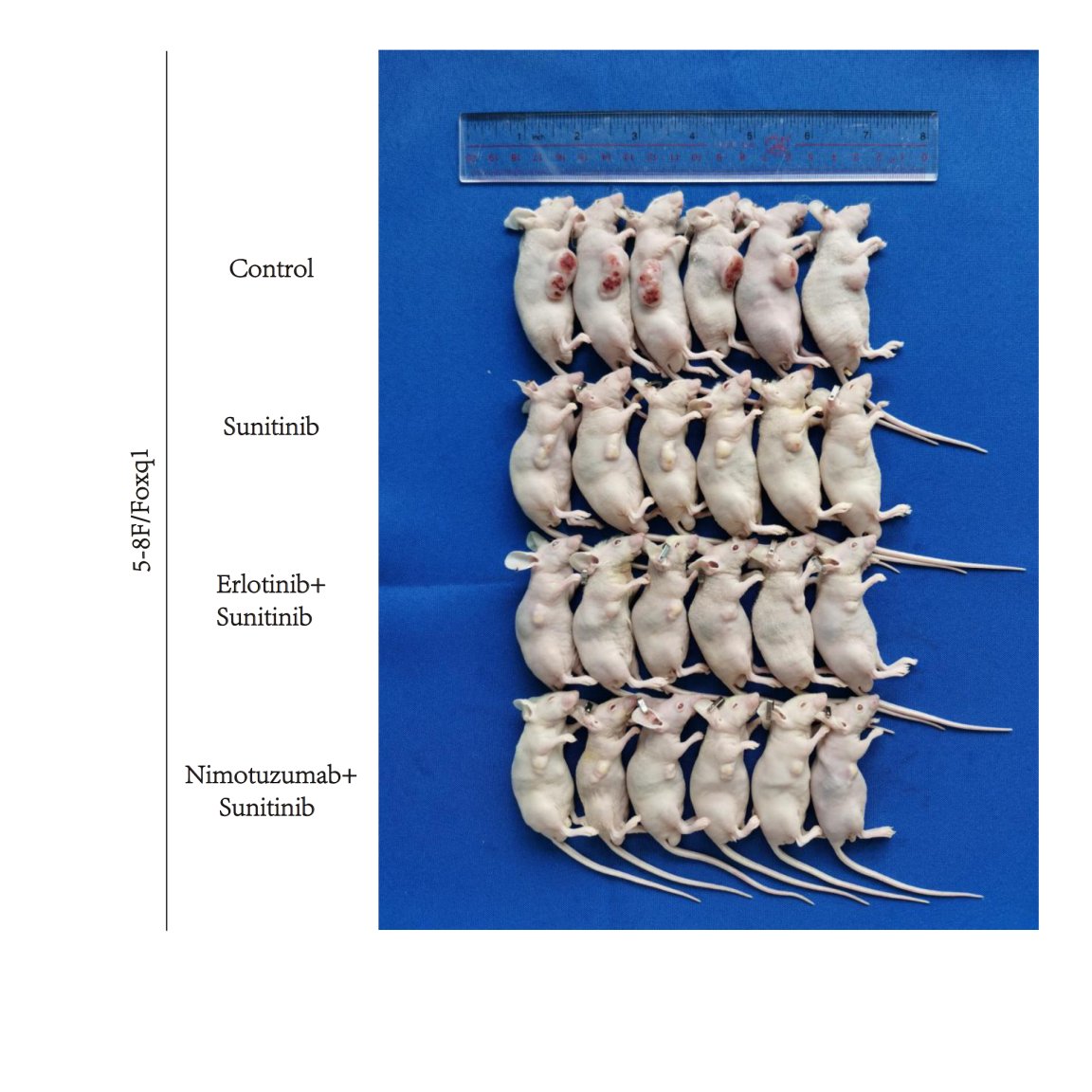


**Figure S3**. Mice were injected subcutaneouslyby Foxq1 overexpressing 5-8F cells, then treated with Sunitinib, combination Sunitinib and Erlotinib, or combination Sunitinib and Nimotuzumab, [respective](file:///I:/数据/投稿文件/cancer%20letters/manuscript%20cancer%20letters.docx#/javascript:;)ly. After 3 weeks, the mice were euthanized.


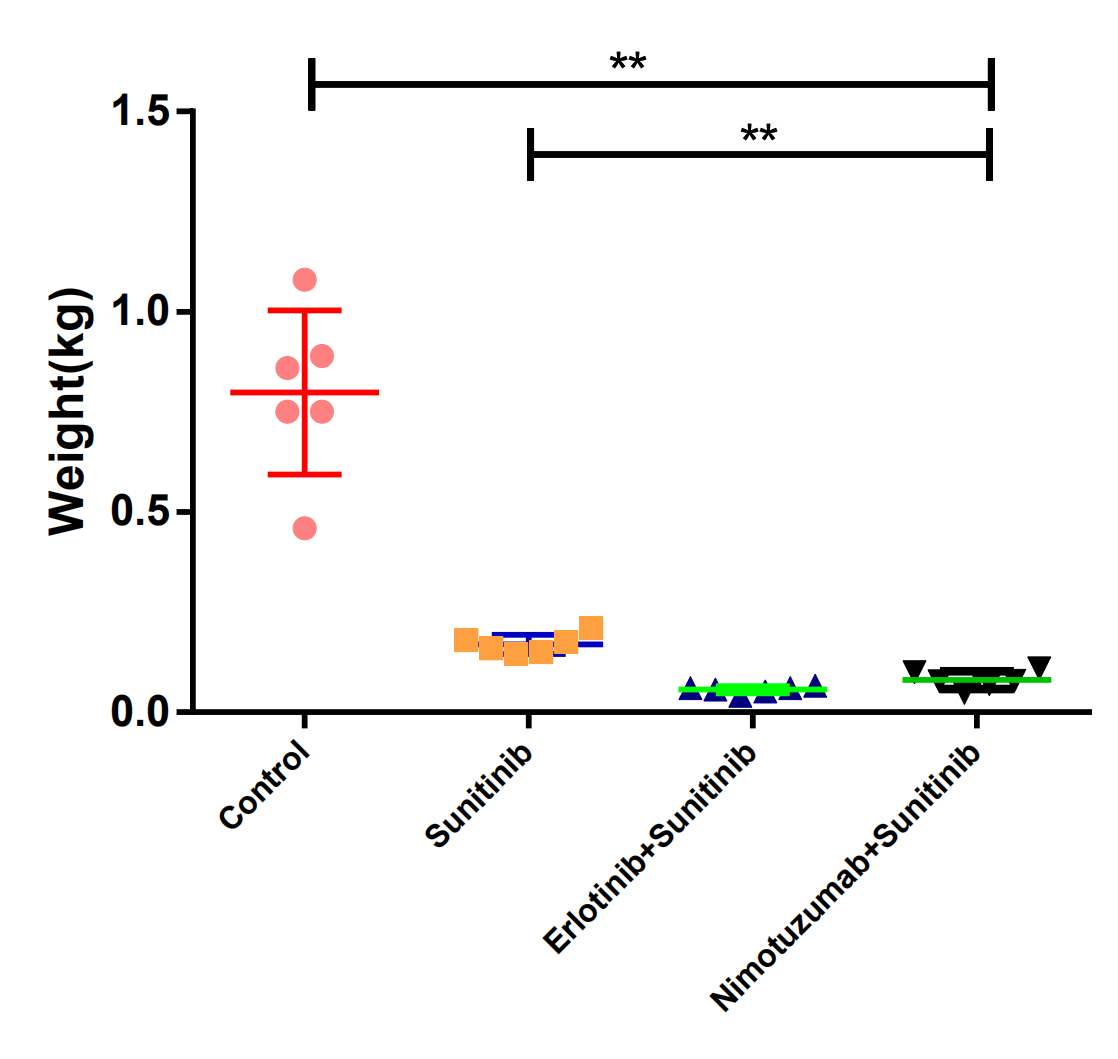


**Figure S4**. The weight of xenografts from each group; p<0.001.


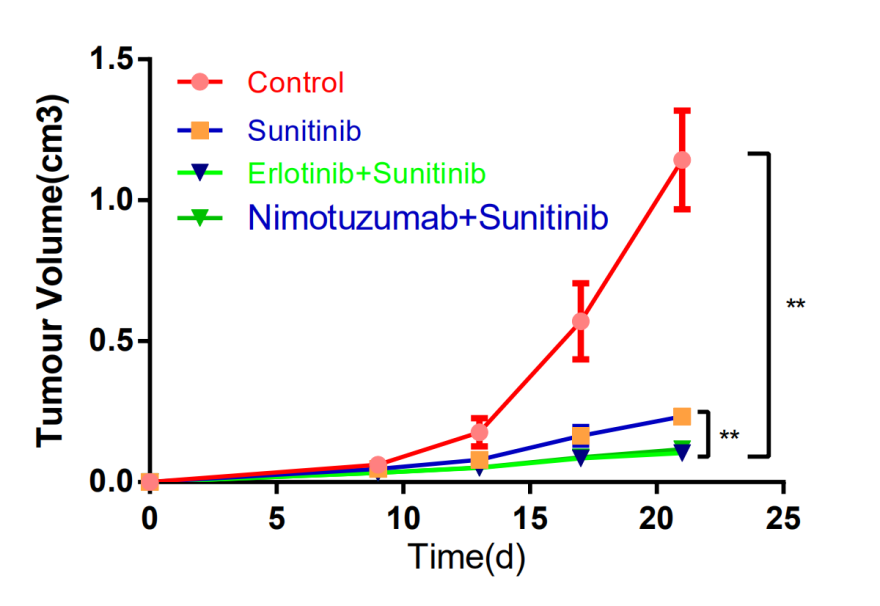


**Figure S5**.The growth curves of the xenograft tumors from each group; p<0.001

**Table S1**. All primers used in this study.

1. qPCR primers:

| CDH5-F | 5' AAGCGTGAGTCGCAAGAATG 3' |
| --- | --- |
| CDH5-R | 5' TCTCCAGGTTTTCGCCAGTG 3' |
| EGFR-F | 5' TTGCCGCAAAGTGTGTAACG 3' |
| EGFR-R | 5' GTCACCCCTAAATGCCACCG 3' |
| AKT-F | 5' TGTGGATTTACCTTATCCCCTCA 3' |
| AKT-R | 5' GTTTGGCTTTGGTCGTTCTGT3' |
| BCL2-F | 5' GGTGGGGTCATGTGTGTGG 3' |
| BCL2-R | 5' CGGTTCAGGTACTCAGTCATCC 3' |
| MMP9-F | 5' TGTACCGCTATGGTTACACTCG 3' |
| MMP9-R | 5' GGCAGGGACAGTTGCTTCT 3' |
| MMP2-F | 5' TACAGGATCATTGGCTACACACC 3' |
| MMP2-R | 5' GGTCACATCGCTCCAGACT 3' |
| GAPDH-F | 5' ACAACTTTGGTATCGTGGAAGG 3' |
| GAPDH-R | 5' GCCATCACGCCACAGTTTC 3' |
| Foxq1-F | 5' CACGCAGCAAGCCATATACG 3' |
| Foxq1-R | 5' CGTTGAGCGAAAGGTTGTGG 3' |
| CLDN1-F | 5' TCTGGCTATTTTAGTTGCCACAG3' |
| CLDN1-R | 5' AGAGAGCCTGACCAAATTCGT3' |
| TEAD1-F | 5' ATGCCAACCATTCTTACAGTGAC3' |
| TEAD1-R | 5' ACAGTTCCTTTAAGCCACCTTTC3' |
| IL6R-F | 5' ATCGGGCTGAACGGTCAAAG3' |
| IL6R-R | 5' GGCGTCGTGGATGACACAG3' |
| SIX4-F | 5' GCATTGAACCCACCAAAAATGT3' |
| SIX4-R | 5' GGAAGTAGACCCCAGTATGTCA3' |
| IRF1-F | 5' CTGTGCGAGTGTACCGGATG3' |
| IRF1-R | 5' ATCCCCACATGACTTCCTCTT3' |
| TCF3-F | 5' CCGACTCCTACAGTGGGCTA3' |
| TCF3-R | 5' CGCTGACGTGTTCTCCTCG3' |
| EHF-F | 5' CAGTGCAGTAGTGACCTGTTC3' |
| EHF-R | 5' CTGTGCTACCATAGTTGGTGTC3' |
| ITGB6-F | 5' GAGGACTACCCGGTGGATTTG3' |
| ITGB6-R | 5' TCCTTTATTGTGTTGAGGTCGTC3' |

1. Dual-luciferase assay primers：

| EGFR F | 5' AAGCTCAAAGGAGAAACTCAAACTTTAC 3' |
| --- | --- |
| EGFR R | 5' CGCTGCTCCCCGAAGAGCTCGCTCCGGC 3' |
| Mutant 1 F | 5' CTTGACAGAAAATATAACGCAAAACCAAATTTGCAGCCCTTGTTAAGTGA 3' |
| Mutant 1 R | 5' GGTTTTGCGTTATATTTTCTGTCAAGGGAATGTGACCTGTGTGTACAAGT 3' |
| Mutant 2 F | 5' GTTGCAATGCGTATTAAGGAGGCCTGTCTCTGCACCCGGAGTTGGGTGCC 3' |
| Mutant 2 R | 5' GGCCTCCTTAATACGCATTGCAACCAGCCATGGGTACTTTGAAGCCAATG 3' |
| Foxq1 F | 5' ATGAAGTTGGAGGTGTTCGTCCCTC 3' |
| Foxq1 R | 5' TCAGGCTAGGAGCGTCTCCACCGGGTAC 3' |

1. CHIP-qPCR primers:

| Mutant 1 F | 5' ACAGGTCACATTCCCTTGACA 3' |
| --- | --- |
| Mutant 1 R | 5' TGTGCATTCACTTAACAAGGGC 3' |
| Mutant 2 F | 5' GGGACCCGAATAAAGGAGCA 3' |
| Mutant 2 R | 5' AGAGCCAGCGTCGGATAATG 3' |
